# Concepts of madness in diverse settings: a qualitative study from the INTREPID project

**DOI:** 10.1186/s12888-016-1090-4

**Published:** 2016-11-09

**Authors:** Alex Cohen, Ramachandran Padmavati, Maia Hibben, Samuel Oyewusi, Sujit John, Oluyomi Esan, Vikram Patel, Helen Weiss, Robin Murray, Gerard Hutchinson, Oye Gureje, Rangaswamy Thara, Craig Morgan

**Affiliations:** 1 Department of Population Health, London School of Hygiene and Tropical Medicine, London, UK; 2 Schizophrenia Research Foundation, Chennai, India; 3 Department of Psychiatry, University of the West Indies, St Augustine, Trinidad; 4 Department of Psychiatry, University of Ibadan, Ibadan, Nigeria; 5 Psychosis Studies Department, Institute of Psychiatry, King’s College London, London, UK; 6 National Institute for Health Research (NIHR) Mental Health Biomedical Research Centre at South London and Maudsley NHS Foundation Trust and King’s College London, London, UK; 7 Centre for Epidemiology and Public Health, Health Service and Population Research Department, Institute of Psychiatry, King’s College London, London, UK

**Keywords:** Psychosis, Cross-cultural research, India, Nigeria, Trinidad, Explanatory models, Help-seeking

## Abstract

**Background:**

In order to facilitate case identification of incident (untreated and recent onset) cases of psychosis and controls in three sites in India, Nigeria and Trinidad, we sought to understand how psychoses (or madness) were conceptualized locally. The evidence we gathered also contributes to a long history of research on concepts of madness in diverse settings.

**Methods:**

We conducted focus group discussions and individual interviews to collect information about how informants in each site make sense of and respond to madness. A coding framework was developed and analyses of transcripts from the FGDs and interviews were conducted.

**Results:**

Analyses suggest the following: a) disturbed behaviors are the primary sign of madness; b) madness is attributed to a wide range of causes; and, c) responses to madness are dictated by cultural and pragmatic factors. These findings are congruent with similar research that has been conducted over the past 50 years.

**Conclusions:**

The INTREPID research suggests that concepts about madness share similar features across diverse settings: a) terms for madness are often derived from a common understanding that involves disruptions in mental processes and capacities; b) madness is recognized mostly by disruptive behaviours or marked declines in functioning; c) causal attributions are varied; and, d) help-seeking is a complex process.

## Background

The pilot study phase of INTREPID was a population-based programme of research designed to implement and evaluate methods for identifying, assessing, and following incident (untreated and recent onset) cases of psychosis and controls in catchment areas in India, Nigeria and Trinidad. Previous publications review our establishment of case-finding networks and our detailed documentation of the nature of mental health systems in the research sites [1], as well as our findings regarding variations in the rates of psychosis and the clinical and demographic characteristics of cases in and across the sites [2]. In this paper we describe our efforts to understand how, in each site, madness is conceptualized. From the perspective of seeking to identify individuals with a psychotic disorder in diverse sociocultural settings, beliefs (or explanatory models) are important, as they will influence who is considered to be suffering from madness, i.e., a condition that might be diagnosed as a psychotic disorder. Consequently, a prerequisite to efforts to identify incident or recent onset cases of psychosis, especially in settings beyond the boundaries of professional mental health services, it was essential to understand how madness was conceptualised locally so that we could explain the nature of our research to informants and, thus, increase the completeness of our case finding [3].

### Explanatory models

In developing the concept of explanatory models as a framework for understanding how individuals make sense of illness, Kleinman [4] suggested a series of questions to elicit such models, including: What name does it have? What are the biggest problems the illness has caused? What caused the problem? What is the appropriate response, treatment? These questions focus on the key pieces of information necessary to elucidate local understandings of illness, i.e., the names or terms used, the signs and behaviours associated with, the causal attributions that help individuals understand, and the local responses when an individual becomes ill with a condition. In regard to mental disorders: a) typical signs range from external, behavioural manifestations to internal, emotional feelings and perceptions; b) causes are often located within a restricted number of domains (i.e., the supernatural or spiritual, the psycho-social, the biological or physical); and, c) responses can occur in one of three sectors of local health care systems (i.e., the popular [e.g., self-help or help from family and friends], the folk [e.g., spiritual and traditional healers], and the professional [e.g., mental health services]) [5]. These formulations shaped how we sought to investigate and understand local concepts of madness in the INTREPID sites in India, Nigeria, and Trinidad.

We use the term *madness* in this paper because, when conferring with informants, we did not use more technical terms. Rather, we began the focus group discussions (FGDs) and individual interviews with examples of behaviours that might be associated with the specific group of disorders, i.e., psychoses, that we wanted to study. Analyses of the FGDs and individual interview transcripts revealed that *mad* and/or *madness* or their equivalents were used in all of the sites. We recognize that the term *madness* may be perceived as derogatory by some. We do not mean to use it that way and would prefer to think of our usage as being in line with other work, e.g., a sweeping history of mental illness [6], a personal narrative of bipolar disorder [7], or an established classic about the establishment of national asylum systems [8].

Through our qualitative research, we generated data that informed our case-finding efforts in the community and provided insights into similarities and differences in how madness is recognized and understood in three economically, socially, and culturally diverse settings.

### Aims

In seeking to understand how, in each site, madness was understood, we sought to investigate two broad questions:

1. How do informants make sense of madness? (i.e., What are the typical signs and behaviours associated with madness? What names/terms do informants use for these signs and behaviours? What are the causes of the typical signs and behaviours?)
2. What do informants believe are the appropriate responses to madness? (i.e., from whom and when should help be sought? What are the appropriate remedies and treatments? How do they relate to causal attributions?)

### Local concepts of madness

Beliefs about health and illness are integral components of local cultures. A large body of research attests to this and shows how such beliefs are shaped by, and in turn shape, health-related behaviours and practices across diverse settings. That is, how individuals, their families and providers make sense of illness influences how they respond to symptoms and behaviours, i.e., from whom help is sought and when, and what types of interventions are obtained [5, 9–11].

In regard to madness, specifically, more than fifty years ago, Edgerton found that violence, aggression and disruptive social behaviours were the most frequent signs of madness among four East African ethnic groups, while hallucinations, the characteristic signs of psychosis in Western psychiatry, were relatively uncommon [12]. Subsequent research outside of Western settings, e.g., in Laos [13], Ethiopia [3, 14], Peru [15], Indonesia [16], China [17] and Uganda [18] generally confirm these findings. A systematic review of explanatory models of psychosis presents a more nuanced perspective [19]. In brief, people often offer multiple and, sometimes, contradictory beliefs about causes. Moreover, large proportions of participants in studies have attributed psychosis to disease (East Africa) [12], social environment (China) [17], psychosocial stress (India) [20], and biological factors (Nigeria) [21]. Edgerton also found that one-quarter of his informants stated they did not know the cause of madness.

Causal attributions have also been associated with help-seeking behaviors. A review by Bhikha and colleagues [19] found that supernatural and psychosocial causal attributions are associated with seeking care from traditional and spiritual healers. Other research suggests that although this may be the case in initial help-seeking [16, 22–24], families may also seek biomedical care [14, 25, 26].

## Method

To investigate how informants make sense of madness and their beliefs about appropriate responses to madness, we conducted FGDs and individual semi-structured interviews with caregivers (relatives), mental health care providers, alternative healers, and key informants. The primary purpose of the study was to inform the development of pamphlets that described, using local terms and understandings of madness, what problems and behaviours were of interest for INTREPID. In other words, this work was conducted to help us develop a shared understanding with providers and informants of the condition for which we were searching.

### Sites

The INTREPID sites (Chengalpet taluk, Tamil Nadu, India; the local government areas Ibadan Southeast and Ona Ara, Ibadan, Nigeria; and, Tunapuna-Piarco municipality, Trinidad) are located in countries that are culturally, socially and economically diverse as evidenced by differing rates of poverty, literacy, and urbanization. Previous publications describe for each of the sites: a) the nature of their mental health systems [1]; and, b) the findings from our pilot study about incidence rates of psychosis [2]. In brief, the sites were distinguished by: a) the presence of numerous spiritual and traditional healers in Ibadan; b) the large number of persons in Chengalpet who had never received biomedical treatments; and, c) readily available professional services in Tunapuna-Piarco but, at the same time, frequent and concurrent reliance on spiritual and traditional healers. Our findings about the incidence of psychosis in the three sites suggest that rates of psychosis, as well as the clinical and demographic characteristics of cases, vary across these populations.

### Samples

In each site, we sought a purposive sample of mental health care providers (e.g., psychiatrists and social workers), healers (e.g., spiritual and traditional), and other informants (teachers, community leaders and workers in non-governmental organizations (NGOs) that formed our case detection system, as well as relatives of individuals with psychosis. Recruitment of informants took place as part of the process of mapping the mental health systems in each site [1]. We used this strategy to ensure that the information we collected came from individuals with a wide range of perspectives about persons with severe mental disorders.

We obtained informed consent from participants in the FGDs and the interviews in the following manner. We approached each potential participant and provided s/he with a copy of our information sheet, which explained that participation was voluntary, that s/he was free to withdraw at any point, without giving a reason, and that all data would be held securely and anonymised. The information sheet was read to non-literate participants and, when it was clear they understood the project, consent was indicated by taking their thumb-print. In Chengalpet, audio consent was obtained from participants in the FGDs.

Locally appropriate compensation was provided to participants.

### Data collection

We used a mixture of FGDs and individual, semi-structured interviews. Our initial intention was to only conduct FGDs. However, this was only possible in Ibadan. In Chengalpet and Tunapuna-Piarco, healers were reluctant to participate in group discussions and, for logistical reasons, it was not possible to hold FGDs with providers in Chengalpet. Consequently, interviews were conducted with individuals from these groups. In all sites, two trained research workers facilitated the FGDs and one trained researcher conducted the interviews. For the FGDs and interviews we used topic guides that included open-ended questions and suggested probes in an effort to concentrate on key domains of interest, i.e., the terms used for madness, as well as the typical signs of, causes of and the responses to madness. FGDs were comprised of only one type of informant and were conducted in a variety of settings. Interviews with healers were conducted in the homes or places of worship; interviews with health care providers were conducted in mental health facilities (see Table 1). FGDs and interviews were conducted by researchers fluent in the local languages. English was used in Trinidad or when participants in the other sites felt more comfortable using English. FGDs and interviews was audio-recorded and transcribed. When necessary, transcriptions were translated into English.

**Table 1:** Number completed focus groups and interviews by type of informant

	Chengalpet		Ibadan		Tunapuna-Piarco	
	FGD (N)	Interview	FGD (N)	Interview	FGD (N)	Interview
Mental health staff	0	8^a^	2 (16)^b^	0	2 (9)^c^	0
Healers, informants	0	6^d,e^	2 (14)^f,g^	0	0	8^h,i^
Relatives	2 (16)^j^	0	2 (13)^k^	0	2 (13)^l^	0

^a^ conducted in offices of several clinics

^b^ conducted at a local government office and a primary health center

^c^ conducted at two primary care clinics

^d^ conducted in homes or places of worship

^e^ 4 spiritual healers; 1 balwadi teacher; 1 panchayat leader

^f^ conducted at two government offices

^g^ 5 spiritual healers; 8 traditional healers; 1 key informant

^h^ conducted in homes or places of worship

^i^ 4 Pentecostal healers; 2 Hindu pundits; 1 Baptist/Orisha healer; 1 Catholic priest

^j^ conducted at mental health outreach clinics

^k^ conducted at University College Hospital and a church

^l^ conducted at two primary care clinics

### Data analysis

We conducted qualitative, exploratory analyses of the transcripts of the FGDs and interviews. First, a designated researcher in each site (RP, SO, and MH) read and annotated three transcripts to identify themes that were suggested, at least in part, by the aims of our project. Second, the annotated transcripts and emerging themes were discussed in detail by all researchers and two study PIs (AC, CM) conferred to clarify commonalities and differences. Third, we generated a coding frame by consensus. Fourth, the designated researchers each applied the coding frame to the same three transcripts, which were then discussed with the two study PIs to ensure consistency in the coding. Fifth, the designated researcher in each site coded the remaining transcripts according to content analysis [27]. Finally, one of the study PIs (AC) carried out the framework analyses [28] using MAXQDA11 qualitative data analysis software (http://www.maxqda.com/).

### Ethical approval

The study was approved by the relevant research ethics committee in each site and in the UK.

## Results

The number of completed FGDs (*n =* 12) and interviews (*n =* 22) by type of informant (relative, professional, healer, informant), in each site is shown in Table 1. The terms used to refer to madness are listed in Table 2. The most prominent themes relevant to our research questions and the number of focus groups and interviews in which they were mentioned, are shown in Table 3.

**Table 2:** Terms for madness

Chengalpet	Ibadan	Tunapuna-Piarco
• gunam marattam (a change in behavior that is not acceptable or is different from usual behaviours) • loosu (a colloquial term-possibly from the English word, loose-meaning can range from someone who is stupid to someone who is mentally ill.) • mad • mana^a^ kolaaru (a problem with the mind) • mana^a^ kulapam/k*ozhapum* (confusion in the mind) • mana^a^ maarudhal (a change of mind in decisions related to any activity) • mana^a^ nalam baathika pattavar (a person whose mental wellness has been affected-often used in context of a precipitating event) • mana^a^ nellai seri illathavar (someone whose state of mind is not alright) • mana^a^ noi (mental illness) • mana^a^ noiali (mentally ill) • manasu kattupai (mental irritation) • manasu seri illathevur (one who is mentally not well) • manavalarchi kundriyavar (one who’s growth of mind is retarded)^b^ • mental • mentally affected • moolai valarchi kummi (poor growth in mental functioning)^b^ • moolai valarchi illathavar (someone who does not have adequate growth of the mind)^b^ • narambhu thalarchi (“nerve weakness,” a term preferable to one that indicates mental illness). • noi vandhirichi (one who is affected by an illness) • paithiyam (colloquial term for being mad/crazy) • pazhuthugal (word most commonly used to imply repairs in machinery. In this context it could mean a reference to the mind being in “repair.”) • psycho (colloquial term)	• abisinwin (going mad after giving birth) • afise (externally induced act or predicament) • aisan (sickness/illness of the brain) • alanganna (a person with odd/abnormal behaviour) • alarun opolo (person with mental disease) • arun apolo (brain sickness) • asinwin (insane/madness) • crazy • didinrin/odoyo (imbecile/fool) • elesimirin (mentally retarded) • mad • ode ori (hunter of the head) • oku oru (in the dead of night) • schizophrenia • iwin (spirit creature) • siwin (being mildly insane) • warapa (epilepsy) • were (mad person/madness) • were kannakanna (mad, psychotic) • were onigbo (madness in which the person wants to be isolated) • ya were tan (gone completely mad)	• altered state • anxiety • attacks • breaking down • challenged • crack up • crazy • curious • demonic possession • depression • dunce • dysfunctional • emotional problem • foolish • going out of mind • gone off • loco • lose their minds • mad • madman • madness • mental disorder • mental problem • mentally sick • mentally unstable • nerves gone bad • not whole within • oppression • psychological problem • schizophrenia • sickness • spiritual problem • spiritually affected • spiritually disturbed/spiritual disturbance • strange • stressed • trip off • eh bête

^a^ mind

^b^ terms refer to persons with less intelligence

**Table 3:** Frequency of responses about Typical Signs, Causes, and Responses & Treatment for madness in each site

		India		Nigeria		Trinidad		Totals	
	Codes	*N*	%	*n*	%	*n*	%	*n*	%
Typical signs									
Somatic	disturbances in sleep, appetite & reports of physical problems	10	10 %	6	6 %	12	9 %	28	8 %
Behavioural	Decline in function								
	not functioning, being unable to work, poor self care	11	11 %	16	16 %	17	12 %	44	13 %
	Isolative								
	withdrawing from interaction	6	6 %	9	9 %	8	6 %	23	7 %
	Self-harm								
	deliberately harming oneself, attempting suicide	4	4 %	4	4 %	6	4 %	14	4 %
	Visibly disturbed (1)								
	violent, assaultive behaviours	19	19 %	26	25 %	19	13 %	64	19 %
	Visibly disturbed (2)								
	being loud, running around, exposing, collecting rubbish	30	29 %	23	23 %	42	30 %	95	28 %
Psychological	loss of interest, sadness, guilt, feeling angry, hostile, etc.	7	7 %	3	3 %	8	6 %	18	5 %
	Suspicions								
	persecutions, referential delusions	7	7 %	3	3 %	2	1 %	12	3 %
	Thoughts & cognition								
	bombarded with thoughts, thought of suicide/killing, problems with memory, concentration	4	4 %	1	1 %	6	4 %	11	3 %
	Hallucinations								
	hearing voices of persons who are not present	4	4 %	11	11 %	21	15 %	36	10 %
	Total	102	100 %	102	100 %	141	100 %	345	100 %
Causes	Don’t know / Other	4	6 %	2	2 %	4	4 %	11	4 %
	Spiritual/Supernatural	26	38 %	24	25 %	57	46 %	107	37 %
	Psychological								
	worry, thinking too much, responses to life events identified as “psychological”	9	13 %	11	11 %	5	4 %	25	9 %
	Social								
	individual’s social environment and social experiences, including family relationships, childhood experiences etc.	12	18 %	17	18 %	24	19 %	53	18 %
	Substance use	1	1 %	16	16 %	13	10 %	30	10 %
	Biological (including genetic)	16	24 %	27	28 %	20	16 %	63	22 %
	Total	68	100 %	97	100 %	124	100 %	289	100 %
Responses & treatment	Folk	57	45 %	73	52 %	130	53 %	260	51 %
	Professional	68	53 %	46	33 %	74	30 %	188	37 %
	Popular	0	0 %	21	15 %	39	16 %	60	12 %
	Total	125	100 %	140	100 %	243	100 %	508	100 %

### Terms

All sites used a wide range of terms for madness (Table 2), many of which had as their root words that meant mad, mind, brain or mental. All sites also associated madness with cognitive impairment and used terms such as *manavalarchi kundriyavar* (Chengalpet), *didinrin* (Ibadan) and dunce or fool (Tunapuna-Piarco). Informants in Ibadan and Tunapuna-Piarco used terms that implied external forces were responsible for a person’s madness, e.g., *afise* (externally induced predicament, Ibadan) and *spiritually disturbed* (Tunapuna-Piarco). In Chengalpet and Tunapuna-Piarco, informants also used terms that may have been less stigmatizing, e.g., *narambhu thalarchi* (nerve weakness, Chengalpet) and *nerves gone bad* (Tunapuna-Piarco). Informants in Chengalpet and Tunapuna-Piarco used colloquial, somewhat pejorative terms for persons who are identified as mad, e.g., *loosu* and *loco*, respectively. Finally, it was only in Ibadan that an informant reported a term (*abisinwin*) that referred to post-natal madness, specifically.

### Typical signs

#### Visible disturbance

In all of the sites, visibly disturbed behaviours were the most frequently reported signs of madness (see Table 3). These behaviours took many forms.

#### Wandering & running away

Wandering and running away were reported in all of the sites. The following statements are illustrative: “My father used to always run out of my home…Once he was missing for two days” (caregiver, Chengalpet); “They can be found at the marketplace, on the highway, under the bridge. The hostile ones are the ones we see roaming the streets and marketplace begging for money” (healer, Ibadan); “One day he start to rant and rave…he running and he screaming and he want to mash up things, ‘cause he hearing voices” (caregiver, Tunapuna-Piarco).

#### Odd behaviour

Odd behaviours, in general, were also considered indicative of madness in all sites, although not consistently. Laughing to oneself or laughing inappropriately was reported in Ibadan and Chengalpet, but not in Tunapuna-Piarco. In contrast, although informants in Ibadan and Tunapuna-Piarco believed that going about naked was a sign of madness, informants in Chengalpet did not associate this with madness. One of the most eloquent statements about odd behaviour was: “But you know, a lot of people they use a vagrant as their reference point for madness. So if you’re on the road, you’re half-naked or your clothes burst down, you’re eating from a dustbin, you’re very dirty or unkempt and so forth, in a lot of the people’s mind that is what madness is” (health care provider, Tunapuna-Piarco).

#### Violence and aggression

Reports of disturbed behaviour were dominated by accounts of violence and aggression, although the levels of violence reported appeared to differ across the sites. In Chengalpet, informants mostly talked about anger and fighting or hitting people, e.g., “He gets very angry, loses temper…We are always scared” (caregiver). In Tunapuna-Piarco, a health care provider reported, “Some patients are very…disruptive in the community. They could be violent…They pelt stones and [treat] people roughly.” In Ibadan, however, descriptions of violence were more extreme, e.g., “A child should not be kept in the same place with a mad person. [If you go] out to wash cloth or you want to go and fetch water and you [leave] a small child, a mad person can decide to throw the child in the river if the river is close to them” (caregiver); and, a healer recounted how a young man with madness got into a quarrel with his mother and “… [pushed her] head in a cooking pot on the fire. Before the mother got to the hospital she died.”

#### Self-harm

Although self-harm, suicide in particular, is often cited as being relatively common among persons with psychosis in many settings [29–32], relatively few informants in the sites linked such behaviour with madness. In Chengalpet, one caregiver and one health care provider mentioned suicide. In Ibadan it was only mentioned in one FGD with caregivers, while in Tunapuna-Piarco suicide was mentioned in one of the caregiver FGDs and in three interviews with healers.

### Decline in function

An obvious decline in function was considered a sign of madness in all of the sites.

#### Poor hygiene

Poor hygiene was reported in all of the sites. The following accounts by caregivers are illustrative. “He might …. decide not to take his bath, change his cloth, and brush his teeth and forget to trim the nails” (Ibadan). “He’s a boy [who] always used to keep himself clean. So, when he started being untidy everybody who knows him knows something wrong” (Tunapuna-Piarco). “My son, does not take bath [and] does not brush his teeth” (Chengalpet).

#### Work performance

Not working or working poorly was associated with madness in all of the sites, especially when considering reports by caregivers. For example: “My son … doesn’t do any work by himself. Only when we ask him to do he will do” (Chengalpet); “Rather than go to his place of work or school, he will leave the house and hide somewhere” (Ibadan); and, “With that schizophrenia thing he doesn’t want to do nothing” (Tunapuna-Piarco). As suggested by a provider in Chengalpet, being “lazy” might reflect the effects of medication, “After treatment they say all is fine. He is not violent anymore which is good but [he] does not do any work. Now when we visit the family, they are telling, ‘He is lazy like a buffalo.’

#### Isolative behaviours

Isolative behaviours were reported in all the sites. For example, a caregiver in Chengalpet stated, “He doesn’t even interact with others,” while healers in Ibadan and Tunapuna-Piarco stated, respectively, “There are some that will not say anything, they will be mute like a stream,” and, “They withdraw from life.”

### Distorted perceptions and beliefs

Distorted perceptions and beliefs were considered signs of madness in all of the sites, but much less frequently than visibly disturbed behaviour. Furthermore, there was variation in the reporting of these signs across the sites. Suspiciousness, talking to self, and lack of “conscious thinking” were reported by informants in all sites, but hearing voices and experiencing visual hallucinations were only reported in Ibadan and Tunapuna-Piarco. For example: “He might see someone and think he is seeing a snake” (health care provider, Ibadan); “My son [looked at] a calendar in our sitting room… [and] suddenly shouted that the picture was talking to him” (caregiver, Ibadan); and, “Once he sees a little child, this voice in his ear says to him, ‘Kill this child’” (healer, Tunapuna-Piarco).

### Somatic symptoms

Although a few informants occasionally mentioned somatic symptoms, e.g., “vomiting continuously,” “severe stomach pain,” and headache, disturbances in sleep were by far the most often identified somatic features of madness. For example: “The mind is disturbed very badly so that they don’t sleep properly, don’t get up on time and does not do their daily chores” (healer, Chengalpet); “Such person may not be able to sleep” (health care provider, Ibadan); and, “Some of them won’t sleep and they would behave very badly” (healer, Tunapuna-Piarco).

### Causes

Informants mentioned a wide range of causes, covering all the domains identified in previous research: supernatural, hereditary (genetic), biological, substance use and psychosocial.

#### Supernatural

Virtually all of the informants in all of the sites mentioned that they or their patients attributed madness to supernatural causes, e.g., black magic, casting of spells, or demonic possession. In Chengalpet, black magic was the most often cited supernatural explanation, e.g., “It’s all black magic. They do it purposefully to destroy the next generation of the family” (healer). In Ibadan, informants spoke about the casting of spells, e.g., “If a child is meant to be great, the evil people are likely to a cast spell on him to hinder him from attaining his goal” (caregiver). Informants in Tunapuna-Piarco also expressed beliefs that madness was the result of being cursed: “People would want to say, is it obeah, is it voodoo, is it black magic?” (healer).

However, psychosis was not always explained as the result of someone invoking supernatural forces as a punishment of an individual or a family. Individuals could also be susceptible to possession by wandering spirits. For example, “It can happen to someone while walking on the street, most especially the pregnant women that are usually warned not to walk in the midday when the sun is high or in the midnight because of those roaming evil spirits” (healer, Ibadan). A healer in Tunapuna-Piarco gave a more complex account, suggesting that psychological states may leave a person open to being possessed by evil spirits: “What we have discovered is that a person moves from the confused state to…the obsessed to the depressed…state. And while that is happening that is really making room for an infiltration of evil powers, evil spirits…We understand from the word of God that Satan is always waiting for an opportunity.”

#### Psychological and social

Psychological and social factors were considered causes of madness in all of the sites and accounted for about a quarter of all causal attributions. The most common of these factors, at least in Ibadan and Chengalpet, concerned overloading the brain, e.g., rumination or “thinking too much”: “If someone thinks too much she will run mad” (caregiver, Ibadan), and, “They must think everything about life. But they have one thought and thinking the same. They participate less and forget about happiness in life” (caregiver, Chengalpet).

Family problems were reported as factors in all three sites and thwarted marriage wishes, tensions from polygamous marriages, and being forced into an unwanted marriage were factors reported in Chengalpet, Ibadan and Tunapuna-Piarco, respectively.

Among the informants in Chengalpet, “shock” was frequently cited as a risk factor, although the nature of shock was not specified. In Trinidad, two informants spoke of traumatic experiences that lead to madness, e.g., “Certain traumatic experiences will be dormant and over the years the person will try to suppress that, but when they see anything that cause a flashback…they will lose control” (healer). Informants in Ibadan did not report trauma as a factor. Instead, they reported disappointment, frustration, and marital problems. It was only in Tunapuna-Piarco that informants mentioned sexual, physical, and emotional abuse, specifically. For example, a caregiver in that site reported, “People who suffer with mental…sometimes they grow with abuse from little children…they’ve been abused, molested. And you growing up with that with your father – your family molest you and every day you living there and seeing them, how you – that would cause a problem mentally – right, psychologically it affecting your brains.”

#### Biological

In all of the sites, familial factors – often vaguely expressed in terms of inheritance – were cited frequently. For example, a healer in Ibadan stated, “If an affected person gave birth to four children, she might be lucky to have two affected children and two normal children. If she gave birth to three children, two might be affected leaving just one free of the illness,” while a caregiver in Trinidad stated, “And it so happened that her brother [was] kind of sick with his brains; so like he inherit[ed] something.” In Chengalpet, genetic attributions were less specific. A key informant suggested that mental illness might result when close relatives marry, and a caregiver observed, “In her family there are several people affected.”

Head or brain injuries were reported in all of the sites as causes of madness, as were fevers, untreated infections and wounds, chemical imbalances in the brain, and brain damage as the result of malnutrition. For example, a health care provider in Ibadan stated, “If somebody has a wound and it is not properly taken care of it might be infected with tetanus thereby leading to brain disorder,” while a healer in that site reported, “If someone has an injury and they do not treat it early, some diseases can come in. It can cause psychosis.”

#### Substance use

In Ibadan and, to a lesser extent, Tunapuna-Piarco, some informants attributed madness to substance use, e.g., cannabis, cocaine, and heroin, e.g., “Another common cause is drug abuse, those that smokes Indian hemp, heroin, cocaine and other harmful drugs” (health care provider, Ibadan); and, “My son was going to school and he started smoking marijuana. And then he got sick and he went to St. Ann’s [the psychiatric hospital]. And then he went to cocaine” (caregiver, Tunapuna-Piarco). In Chengalpet, only one care provider mentioned drug use, and only one key informant cited alcohol abuse as causes of madness.

#### Multiple causes

Many informants cited multiple causes of madness. A healer in Chengalpet not only attributed madness to black magic and evil spirits but also men being driven mad by the infidelity of women. Similarly, a spiritual healer in Tunapuna-Piarco mentioned several supernatural causes of madness, but also spoke of sexual abuse in childhood and being forced into unwanted marriages as psychosocial causes of madness. Members of an FGD of healers in Ibadan spoke of womanizing and thinking too much as causes. Caregivers in Ibadan also cited eating unwholesome foods, “ancestral curse,” and hereditary. One caregiver stated, “Psychosis can be caused by many things.” while another caregiver in the same FGD agreed. Interestingly, neither seemed to give more weight to one cause or another.

There was little evidence that the various causes had multiplicative effects, although one spiritual healer in Tunapuna-Piarco believed that supernatural causes functioned in the presence of psychological vulnerability: “Satan is always waiting for…opportunities to destroy individuals and he can only do so when the person is weak mentally.”

### Responses to madness

Our coding scheme for responses to madness followed Kleinman’s model of health care systems [5] and included the following categories: professional, folk and popular, with several sub-categories in each. Our findings suggest that responses are best described by narratives of dynamic help-seeking. That is, initial responses do not necessary predict responses to subsequent episodes, which appear to be varied and based on pragmatic issues.

The most frequent response to madness was to seek help in the folk sector, which in all three sites was comprised of spiritual and traditional healers: Hindu temples, Muslim shrines, numerous small Christian churches in Chengalpet; spiritual healers associated numerous churches and traditional healers who employ herbs, roots, oils and sacrifices in Ibadan; and, in Tunapuna-Piarco, spiritual healers from a wide range of religions, as well as practitioners of *obeah* who perform rituals to counteract curses and spells (Table 3) [1].

Seeking professional help was the next most frequent response. In Chengalpet this meant: a) going to a variety of hospitals and private psychiatrists in the catchment area or in the nearby city of Chennai; b) attending primary care centers that participate in the National District Mental Health Program; or, c) seeking care in psychiatric clinics that are operated by two non-governmental organizations. In Ibadan, there are no professional services within the catchment areas and care may be sought in one of three hospitals located elsewhere in the city. In Tunapuna-Piarco, two primary care centers provide regular psychiatric clinics. In addition, one private and one public hospital offer mental health services. The main psychiatric hospital in Trinidad is located outside of the catchment area but it provides services to residents of Tunapuna-Piarco [1].

Respondents infrequently described efforts to manage madness within the family or the popular sector. This may reflect the nature of questions asked, which probably made respondents think in terms of external agencies. However, caregivers in Ibadan reported chaining as a first step, e.g., “Once it is suspected that somebody has this sickness, call the men in the house to chain the patient, after that barb the hair then lock him up.” At the same time, caregivers urged, “Anybody that has this problem should be showered with care from people around them.” In contrast to Ibadan, where informants spoke of prayer, fasting and the use of herbs and oils in the context of seeking help in churches or from traditional healers, caregivers in Tunapuna-Piarco spoke of using these methods in more personal ways, e.g., “I does pray a lot…Every 1 o’clock, 2 o’clock at night I does get up and say my Psalms and pray for him. And I think that helps a lot.” The transcripts do not allow us to determine whether this contrast between the sites represents differences in behaviours or differences in styles of reporting.

Reports about how the early stages of madness were managed within the popular sector were absent in Chengalpet, but there is a need for caution: it is possible this topic was not adequately addressed in the focus group discussions. Furthermore, since a majority of cases in Chengalpet had not received biomedical treatment for many years, it seems logical to conclude that families were managing most care on their own.

This summary of the specific responses to madness does not, however, fully convey caregivers’ complex narratives of what they did or what they believed should be done in response to the emergence of madness in a family member. In Chengalpet, caregivers described how they had for years and, at great expense, sought treatment from spiritual healers, e.g., “I took to all places, Sir. I lost all jewels and cash for those things and don’t have even a single penny in hand.” As the multiple spiritual treatments failed many caregivers came to rely on “English medicine” (i.e., injections and tablets of antipsychotics): “Want to take them to hospital only, Sir. It gets cured only by taking to hospital and by taking tablets.” This does not tell the whole story, however. Caregivers also believed that medicines would not work if madness was due to black magic, e.g., “If it is black magic then when we go to the temple and pray it will be alright. If that doesn’t cure the illness then it means that there is some problem in the body.”

In Ibadan, where we held one caregiver focus group in a church and one in University College Hospital, reports varied about responses to madness. Members of the church group expressed skepticism or disbelief in the effectiveness of biomedicine, e.g., “It is not all madness that injection can treat.” Another caregiver asserted, “In the olden days everybody knows they didn’t take such problem to the hospital… [they went to] traditional healers and church for treatment.” Nevertheless, some informants suggested that going to the hospital was a good idea because, for example, “[the patient should be] given an injection so that he can sleep and his brain be settled.”

In contrast, caregivers in the focus group at University College Hospital believed in biomedicine and cautioned against believing the claims of spiritual and traditional healers: “When you are not well informed you make a lot of mistakes. [In the churches] they will tell you going to the hospital is a mere waste of time. You will just be going from place to place where they will be extorting money from you. It is a foolish step caused by ignorance. But if one is lucky enough to discover the hospital route on time one will get solution.” Other caregivers in the group described how they had sought spiritual treatment but that the ill family member kept relapsing: “The first step we took was to go to a prayer mountain [an apostolic or evangelical church], but the treatment relapsed. When we are there she will stabilize but as soon as we get back to the house the sickness will increase. There was a day we went to [another prayer mountain and] we noticed that she was a little bit better but as soon as we got home the sickness started again…We were later referred to UCH [University College Hospital] here in Ibadan.” The views of caregivers in this group might be summarized by: “If you can go directly to the hospital there will be a quick breakthrough…The best step is the hospital.”

The caregiver focus groups in Tunapuna-Piarco also depicted complex accounts of responses to madness. One informant offered, “I went to the clinic first and then…carried him to church. Then I decided that I would get somebody to clean the house spiritually.” Other caregivers reported using herbs, fasting, bathing the ill member with oils, prayer, and seeking the help of “spiritual people.” At the same time, some caregivers reported reliance on biomedical care and frequent hospitalization, although this may be, at least in part, an artifact of the focus groups being held in two primary care clinics that provided regular specialist psychiatric care. Nevertheless, the evidence of reliance on prayer, herbal remedies, and biomedical care indicates that caregivers resorted to multiple treatment strategies.

### Concepts according to type of informant

Analyses suggest that, in each site, caregivers, health care providers and healers were in general agreement in their reports of local concepts of madness, i.e., typical signs of, responses to and causes of madness.

## Discussion

The findings reported above are consistent with previous research conducted in or near the INTREPID sites. Research in southeastern Nigeria [21] found a predominance of supernatural causal attributions of madness, but that biological (e.g., head injury and substance abuse) and psychosocial factors were also important. A 1998 study in Chengalpet found highly complex beliefs about the causes of madness, e.g., family conflicts, financial problems, alcohol abuse, and supernatural causes [25]. To the best of our knowledge, only one study in Trinidad has examined this topic and it found that about one-quarter of medical students attributed psychosis to supernatural causes [33].

Our findings are also consistent with a systematic review by Bhikha and colleagues, which found that a majority of people in developing countries have supernatural or psychosocial causal attributions for psychosis [19]. We also found, as did the review, that people often held multiple and sometimes contradictory beliefs about causes. However, we found that biological and substance use explanations for madness (22 and 10 %, respectively) were relatively frequent among the participants in our study, whereas these causes are hardly cited in the review by Bhikha and colleagues. Moreover, our study suggests that together biological and substance use causal attributions for madness made them more prominent than psychosocial and almost as prominent as supernatural causal attributions.

Results of individual studies included in the review by Bhikha and colleagues suggests there is significant variation in beliefs about the causes of psychosis in non-Western settings. For example, large proportions of participants in studies have attributed psychosis to disease (East Africa) [12], social environment (China) [17], psychosocial stress (India) [20, 25], and biological factors (Nigeria) [21]. The INTREPID research found that all of these factors were cited by participants in the three sites. In contrast, whereas Edgerton’s research found a large proportion of informants who did not know the cause of madness, this was an uncommon response among the INTREPID informants.

The review by Bhikha and colleagues [19] found that supernatural and psychosocial causal attributions were associated with seeking care from traditional and spiritual healers. Other research suggests that although this may be the case in initial help-seeking [16, 22–24], families often also seek biomedical care [14, 25, 26], and previous research in Ibadan [34] and southern India [35] has found that help-seeking was not necessarily determined by notions of causal attribution. Indeed, our research suggests that patterns of help-seeking are complex. Although causal attributions may influence initial responses, over the long-term, pragmatic considerations, e.g., effectiveness of treatments, accessibility of services, and treatment costs, become prominent.

One surprising non-finding concerned the infrequency of reports about self-harm or suicide, which have been cited as prevalent among persons with psychosis e.g., [30, 36–38]. For example, a 25-year follow-up of 90 persons with schizophrenia in Madras (now Chennai) found that 32 % of mortality was due to suicide and a large proportion of the cohort had attempted suicide [39]. It is possible that the relative infrequency of reported self-harm and suicide in INTREPID was due to sociocultural proscriptions against talking about suicide, however this is a question that awaits further investigation.

### Methodological issues

Several methodological issues limit the extent to which it is possible to generalize from these data. First, our primary purpose in collecting these data was to inform and facilitate the identification of individuals with an untreated or recent onset psychotic disorder. As a consequence, we centered our selection of individuals for the FGDs and interviews within the case-finding systems we developed in each site for the purpose of detecting persons with an untreated or recent onset psychotic disorder [1]. It might be expected that this sampling strategy would over-include those with beliefs that were mostly in line with those embodied in biomedical models of psychosis. However, the inclusion of many healers and caregivers meant that a wide range of perspectives was represented.

Second, health care providers were not asked about their understandings of madness. Rather, they were asked about how people in their communities understood and recognized madness. This meant their responses might be a biased view of the understandings of the broader community. However, the results of the analyses do not suggest any systematic biases in this regard.

Because of local constraints, we conducted both FGDs and individual interviews. This is problematic for a number of reasons, not least that the dynamics of each can produce different data. For example, focus groups tend to encourage consensus and can obscure alternative opinions and beliefs. At the same time, individual interviews may tap into a wider range of beliefs, but this may also give undue prominence to relatively idiosyncratic ideas that are not necessarily shared by many others within the wider culture. The patchwork of focus groups and interviews that we were able to complete within and across the sites, consequently, urges caution in inferring too broadly from our findings about beliefs common in each of the settings.

## Conclusions

The findings from the INTREPID project suggest that concepts of madness have similar features across diverse settings: a) terms for madness are often derived from a common understanding that the causes of madness can be located in disruptions of mental processes and capacities; b) madness is recognized mostly by disruptive behaviours or marked declines in functioning; c) causal attributions are varied and include, for example, supernatural and biological factors; and, d) help-seeking is shaped by causal attributions, as well as economic factors and assessments of treatment effectiveness. In addition, our findings are consistent with research findings that go back to Edgerton’s work in the 1960’s [3, 12–21].

This is not to say, however, that one can ignore variations across the sites. For example: a) although informants in Ibadan and Tunapuna-Piarco reported that going about naked was a sign of madness, informants in Chengalpet did not; b) in Ibadan informants spoke of prayer, fasting and the use of herbs and oils in the context of seeking help in churches or from traditional healers, while caregivers in Tunapuna-Piarco spoke of using these methods in more personal terms; and, c) the use of cannabis as a cause of madness was often cited in Ibadan and Tunapuna-Piarco, but not in Chengalpet.

In conclusion, knowledge of these similarities and the nuances of differences are key to understanding how local beliefs and material conditions influence how people understand, recognize and respond to madness. In turn, this knowledge of local concepts is essential when searching for psychosis in diverse places and makes possible the collection of “comparable epidemiological cohorts” of first-episode and untreated cases of psychosis in settings where our knowledge of psychosis is limited and professional mental health services are not always the primary source of care for people with psychosis [40]. Last, we believe our findings make a significant contribution to a long history of research about concepts of madness in diverse settings.
